# Detection, Purity Analysis, and Quality Assurance of Adulterated Peanut (*Arachis Hypogaea*) Oils

**DOI:** 10.3390/foods7080122

**Published:** 2018-07-31

**Authors:** Shayla C. Smithson, Boluwatife D. Fakayode, Siera Henderson, John Nguyen, Sayo O. Fakayode

**Affiliations:** Department of Physical Sciences, University of Arkansas Fort Smith, 5210 Grand Avenue, P.O. Box 3649, Fort Smith, AR 72913-3649, USA; ssmith10@g.uafs.edu (S.C.S.); Bolufakadami@gmail.com (B.D.F.); shende00@g.uafs.edu (S.H.); jnguye00@g.uafs.edu (J.N.)

**Keywords:** peanut-oil, food-analysis, peanut-oil-adulteration, infrared-spectroscopy, partial-least-regression-analysis, food-quality-assurance

## Abstract

The intake of adulterated and unhealthy oils and trans-fats in the human diet has had negative health repercussions, including cardiovascular disease, causing millions of deaths annually. Sadly, a significant percentage of all consumable products including edible oils are neither screened nor monitored for quality control for various reasons. The prospective intake of adulterated oils and the associated health impacts on consumers is a significant public health safety concern, necessitating the need for quality assurance checks of edible oils. This study reports a simple, fast, sensitive, accurate, and low-cost chemometric approach to the purity analysis of highly refined peanut oils (HRPO) that were adulterated either with vegetable oil (VO), canola oil (CO), or almond oil (AO) for food quality assurance purposes. The Fourier transform infrared spectra of the pure oils and adulterated HRPO samples were measured and subjected to a partial-least-square (PLS) regression analysis. The obtained PLS regression figures-of-merit were incredible, with remarkable linearity (*R*^2^ = 0.994191 or better). The results of the score plots of the PLS regressions illustrate pattern recognition of the adulterated HRPO samples. Importantly, the PLS regressions accurately determined percent compositions of adulterated HRPOs, with an overall root-mean-square-relative-percent-error of 5.53% and a limit-of-detection as low as 0.02% (*wt/wt*). The developed PLS regressions continued to predict the compositions of newly prepared adulterated HRPOs over a period of two months, with incredible accuracy without the need for re-calibration. The accuracy, sensitivity, and robustness of the protocol make it desirable and potentially adoptable by health departments and local enforcement agencies for fast screening and quality assurance of consumable products.

## 1. Introduction

An increase in world population and industrial development has resulted in high demand for consumable products including edible oils. Edible oils are used for domestic cooking, deep frying in fast food restaurants, and for other industrial applications [1,2]. Edible oils are composed of triglyceride molecules, which are required, in certain amounts, in the human diet for energy production and energy storage [3,4]. However, high demand for edible oils such as highly refined peanut oils (HRPOs) has resulted in adulteration of edible oils with cheap, unhealthy, or synthetic oils. Municipalities, health departments, and regulatory agencies, including the United States Food and Drug Administration (FDA), the United State Department of Agriculture, the European Commission, the European Food Safety Authority, and the World Health Organization, are relentless in their efforts to curtail the sales of fake, substandard, and/or adulterated consumable products [5,6,7,8,9,10,11,12,13,14]. For instance, efforts have been made by FDA and World Health Organization to prohibit the sale of unhealthy oils and to eliminate trans-fats in the human diet by 2018 and 2023 [15]. Nonetheless, a significant percentage of all consumable products, including edible oils, are neither screened nor monitored for quality control and quality assurance for various and diverging reasons.

Counterfeiting and/or the adulteration of consumable products is even more problematic, rampant, and worrisome in developing countries, where most regulatory agencies lack the infrastructure, skilled inspectors, and/or financial resources to enforce the screening of consumable products. The loopholes and deficiencies in the global monitoring scheme make numerous consumable products highly susceptible to and easy targets for adulteration and/or trafficking. The prospective adulteration of edible oils raises concern about the production of safe edible oils for human consumption. The potential intake of fake and adulterated oils and its associated health impacts are also a nightmare, raising a public health safety concern. For instance, the intake of adulterated and unhealthy oils, and trans-fats in the human diet has had negative health repercussions, including cardiovascular disease, causing millions of deaths annually [15]. 

To address these concerns, efforts have been devoted to the development of analytical strategies including the use of a high performance liquid chromatography (HPLC), mass spectrometry, electronic nose, isotopic dilution, biomarkers and sensors, nuclear magnetic resonance, deoxyribonucleic acid (DNA) barcoding, and electroanalytical techniques for quality control and assurance of consumable products and edible oils [16,17,18,19,20,21,22,23,24,25,26,27,28,29]. Regardless of the high sensitivity and good accuracy of these techniques, they have inherent challenges and drawbacks such as long analysis times, high cost of instrumentation, and required special training. In addition, some of these methods are not portable, limiting their wider applicability for routine in situ field screening of consumable products. Raman and infrared spectroscopy are non-destructive and rapid techniques that require a small sample size and are capable of solid and liquid sample analysis with little or no sample preparation, making them ideal for fingerprinting, determination of authenticity, and quality assurance of consumable products including edible oils [30,31,32,33,34,35,36,37,38,39,40,41,42,43,44,45,46,47,48,49,50]. Besides, Raman and infrared spectrometers are portable and fairly inexpensive, allowing affordable and fast in situ field screening of consumable products. Moreover, a combined use of molecular spectroscopy, including Raman, infrared, and fluorescence spectroscopy, and multivariate analyses, has increasingly been used in recent years for sample and instrument calibrations, purity analysis, and quality assurance of consumable products [30,31,32,33,34,35,36,37,38,39,40,41,42,43,44,45,46,47,48,49,50]. 

Research from our laboratory [51,52] and from other research laboratories [30,31,32,33,34,35,36,37,38,39,40,41,42,43,44,45,46,47,48,49,50] has revealed the potential utility of the combined use of molecular spectroscopy and multivariate regression analysis for food purity analysis and quality assurance of adulterated edible oils and essential oils. However, numerous edible oils of high dietary importance and market values such as highly refined peanut oil (HRPO) that are susceptible to adulteration and/or trafficking are yet to be investigated. This study reports a simple, fast, sensitive, accurate, and low-cost chemometric approach to the quality assurance of HRPOs that were adulterated either with edible vegetable oil (VO), canola oil (CO), or almond oil (AO). Specifically, the combined use of Fourier transform infrared spectroscopy (FTIR) and multivariate partial-least-square (PLS) regression for detection, purity analysis, and quality assurance of adulterated HRPOs was investigated.

Peanut oil is derived from the peanut (*Arachis hypogaea*), a legume that is rich in proteins, vitamins, phytochemicals, anti-oxidants, polyphenols, polyunsaturated, and fiber [53,54,55,56]. In addition to edible oil production, peanuts have a wide range of other industrial utilities, including the production of peanut butter, peanut flour, animal feed, groundnut cakes, animal protein supplements, and poultry rations [55,57]. The global production of peanut oil is estimated at 5.88 million metric tons in 2018 with multimillion dollar annual global peanut oil sales [55,57]. Highly refined peanut oil is a healthy choice and is widely used for domestic cooking, deep frying in fast foods restaurants, and as salad oils around the world. For instance, Chick-fil-A, one of the leading North American fast-food restaurants, with approximately 2200 restaurants in USA and Canada with $8 billion dollar in revenue, only uses 100% refined peanut oil for all of its cooking and deep frying. Other notable fast-food restaurants including Five Guys, Jimmy Johns, and Subway only use HRPO on their French fries, kettle cooked chips, and carved turkey, respectively. Highly refined peanut oil undergoes several industrial processes including the extraction of protein allergen, discoloration through bleaching, and deodorization [55], making it relatively more expensive than the crude peanut oil. The sale of fake-peanut oil and adulterated HRPO with edible vegetable oils or synthetic oil with a peanut aroma is quite frankly a global challenge, causing economic losses to producers of authentic HRPO.

## 2. Materials and Methods

### 2.1. Material and Supplies: Regression Analysis

Highly refined (100%) peanut oil (HRPO) and adulterant vegetable oil (VO), canola oil (CO), and almond oil (AO) were purchased from a local grocery store in Fort Smith, Arkansas, USA.

### 2.2. Preparation of Adulterated HRPO Samples, FTIR Measurement, PLS Regression, and Multivariate Data Analysis

Twenty-five training sets and calibration samples of adulterated peanut samples were used for each study conducted with vegetable oil, canola oil, and almond oil adulterants. The training set and calibration samples (*n* = 25) of varying compositions of adulterated HRPO with either VO, CO, or AO, ranging from 1–90% (*wt/wt*), were prepared in sample vials. The samples were kept at room temperature for approximately 48 hours to facilitate homogenization of HRPO and the adulterant oils. The FTIR spectra of the adulterated HRPOs were measured using an ATR-FTIR spectrometer (Thermo Scientific NiCOLET iS5, Waltham, MA, USA). The FTIR spectrum of each sample was scanned 25 times with a resolution of 4 cm^−1^ over a 600 cm^−1^ to 4000 cm^−1^ wavenumber range. Partial-least-regression and chemometric data analysis was performed using the software The Unscrambler (CAMO Software, 9.8, Oslo, Norway).

## 3. Results and Discussion

### 3.1. Physical Examination and FTIR Property of Pure and Adulterated HRPO Oils

The initial study involved the physical and FTIR spectroscopic examination of pure edible highly refined peanut oil (HRPO), vegetable oil (VO), canola oil (CO), almond oil (AO), and adulterated HRPOs. Highly refined peanut oil is pale-yellow, with no apparent peanut odor. The physical appearance and the color of pure HRPO, VO, CO, and AO are very similar and indistinguishable. Similarly, the physical appearance, including the color, of pure HRPO and adulterated HRPOs counterparts is identical, making it challenging to use ordinary visual examination for the detection of a suspected adulterated HRPO. 

**Figure 1:**
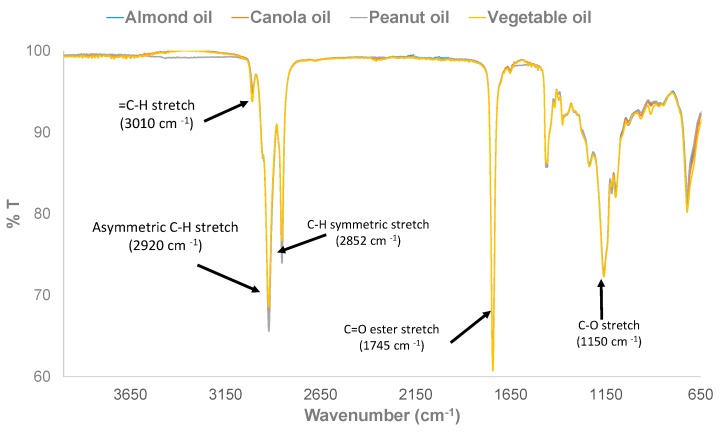
FTIR spectra of pure highly refined peanut oil, vegetable oil (VO), canola oil (CO), and almond oil (AO).

**Figure 2:**
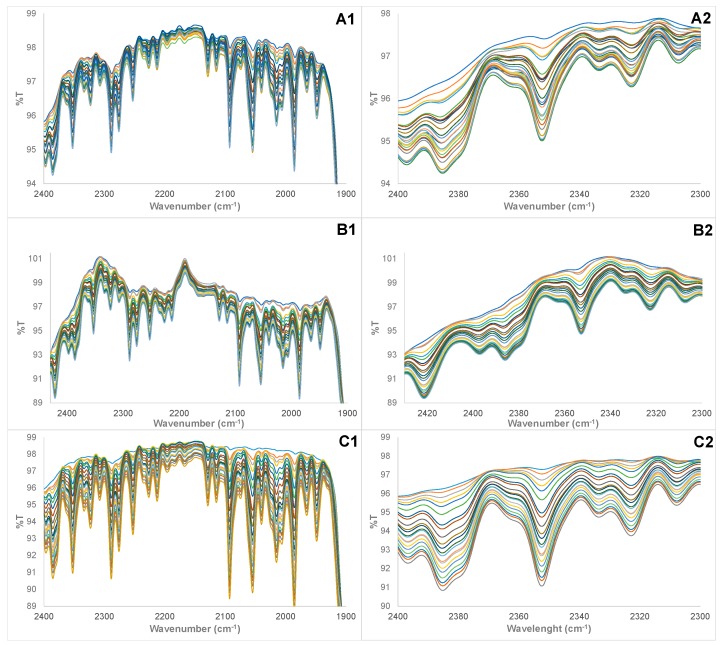
Cross section of FTIR spectra of the training set and calibration samples of: (**A1**,**A2**) highly refined (100%) peanut oil (HRPO) adulterated with vegetable oil, (**B1**,**B2**) HRPO adulterated with canola oil, (**C1**,**C2**) HRPO adulterated with almond oil.

The FTIR spectra of pure HRPO, VO, CO, and AO samples showing the notable and characteristic C–CH_2_ asymmetric stretch (C–H) stretching (~2921 cm^−1^); CH_2_ symmetric stretching (C–H) (~2853 cm^−1^); ester C=O stretching (~1745 cm^−1^); CH_2_ wagging (~1160 cm^−1^); symmetric H–C–H bending (~1380 cm^−1^); and CH_2_ scissoring (~1460 cm^−1^) of triglyceride component of HRPO, VO, CO, and AO [32,33] are shown in Figure 1. Expectedly, pure HRPO, VO, CO, and AO have similar FTIR absorption profiles, primarily because all edible oils contain the triglyceride molecules that are responsible for FTIR absorptions [32,33]. Also, edible oils contain triglyceride molecules that are required, in certain amounts, in the human diet for energy production, utility, and energy storage [3,4]. Figure 2 shows the cross sections of FTIR spectra of the training set and calibration samples with varying % composition of HRPO adulterated with VO, CO, and AO adulterants. Although the physical appearance of the pure HRPOs and adulterated HRPOs are indistinguishable, the profile of FTIR spectra of pure HRPO and adulterated HRPO differ and vary with the percentage compositions of the adulterated HRPO samples. The observed variations and changes of FTIR spectra with compositions of adulterated HRPOs is an indicative of interactions of the HRPO with adulterant oils as a result of hydrophobic interactions and/or through hydrogen bonding involving the triglyceride carbonyl group. Differences in the FTIR spectra profile of pure HRPO and adulterated HPPO can, therefore, be used for quick screening for the detection of adulterated peanut oils.

### 3.2. PLS Regression Modeling

The complexity, variation, and spectral overlapping at multiple wavenumbers observed in Figure 2 preclude the likely use of ordinary visual examination for adulterated HRPO pattern recognition or the use of univariate spectral analysis (spectral analysis at one wavenumber) to achieve any meaningful sample calibrations or regression analysis for purity analysis and determination of percent composition of adulterated HRPO samples. The use of multivariate analysis (spectral analysis over a range of wavenumbers) such as partial-least-square (PLS) is more desirable and capable of complex spectral data analysis for sample calibration. The PLS can capitalize on the changes and variability, such as those observed in Figure 2, to extract the most valuable information that is required for sample calibration and for PLS regression modelling to determine the compositions of adulterated HRPOs. The most valuable information in the spectral data set is invariably accompanied with the directions that contains the most substantial variability. The detailed PLS mathematical expressions have been comprehensively discussed and reported elsewhere [58,59,60,61,62].

Generally speaking, the goal of any PLS regression is to decompose the original data matrix A into two components, a “*structure component*” and a “*noise component*” that can be represented by Equation (1).

*A* = *TP^T^* + *E* (1)

where *A* is the original *k* × *n* data matrix of FTIR % transmittance data of adulterated HRPOs in this study, *T* and *P* are two new matrices that must be evaluated and determined, and *E* is a *k* × *n* residual matrix that represents the unexplained variance or “*noise component*” in the model. The “*structure component*” of *A* is given by *TP^T^* where the superscript *T* denotes the transpose of *P*, achievable by substituting rows for columns. Each PLS component is a variance-scaled vector that accounts for a certain amount of variability in the data set.

Partial-least-regression modelling also aims to determine a regression vector, which constitutes the mathematical model that relates the FTIR spectral data in this study to the % compositions of adulterated HRPOs. In the case of a single sample, the relationship between the dependent variable (*y*-variable, % composition of adulterated HRPOs) and the independent variable (*x*-variables, the FTIR spectral data) can be expressed mathematically using Equation (2).

*y*_i_* = b*_0_* + x*_1_*b*_i1_*+ x*_2_*b*_i2_* + x*_3_*b*_i3_*+*……… *x_n_b*_i*n*_ (2)

where *y*_i_ is the value of *y* predicted by the PLS regression model for the ith sample, the *b*_i_ are the regression coefficients that constitute the regression vector, and the *x*_iλ_ terms represent the FTIR intensities for the ith sample over the wavenumber index from 1 to *n*. Equation (2) can further be expressed in matrix notation as shown in Equation (3).

*Y* = *Xb* (3)

where *Y* contains the matrix values of the dependent variables for all samples, *X* is a matrix composed of values of the independent variables of all samples, and *b* contains the regression vector. As soon as a regression model has been established and optimized, it can be utilized to calculate *y*_i_ for any series of unknown samples exclusively from their spectra using Equation (3).

The predictive ability of any PLS regression model for the *y*-variable invariably relies on the assumption of no co-linearity among the *x*-variables, which is an invalid assumption for a PLS regression involving spectral data analysis. Hence, the initial task in any PLS regression modeling is to carefully eliminate any inherent co-linearity in the spectral data or *x*-variables. Removal of co-linearity among *x*-variables is achievable by transforming the original data matrix **A** from the initial *xyz*-coordinate system (made up of *n* variables) into a new variance-scaled eigenvector coordinate system with fewer variables, where each new variable is orthogonal to the others. The use of PLS is desirable because it reduces the data dimensionality from *n* to a significantly smaller value. Additionally, the actual number of vectors required to construct the new variable space is adjustable to fit the expected “*noise*” level of the original data matrix **A**. The new variance-scaled eigenvector coordinate system is thus composed of a smaller number of orthogonal vectors known as the partial least square (PLS) component. The first PLS component of a dataset usually accounts for most of variance in the data. Each successive PLS component accounts for a lesser variance in the dataset. Therefore, only a few PLS components often contain the most valuable information in a dataset. After the first few PLS components are evaluated and determined, the remaining variance is summed together into the ***E*** matrix (noise component) that is not accounted for by the PLS model is eliminated.

### 3.3. Figures-of-Merit of PLS Regression Model, Limit-of-Detection (LOD), and Limit-of-Quantitation (LOQ)

**Figure 3:**
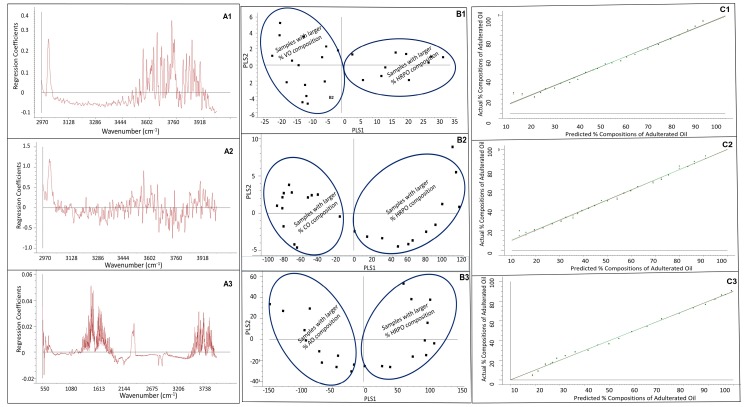
Summary of the partial least square (PLS) regression: (**A1**) regression coefficient of PLS versus wavenumber of HRPO adulterated with vegetable oil; (**A2**) regression coefficient of PLS versus wavenumber of HRPO adulterated with canola oil; (**A3**) regression coefficient of PLS versus wavenumber of HRPO adulterated with almond oil; (**B1**) score plot of PLS regression of HRPO adulterated with vegetable oil; (**B2**) score plot of PLS regression of HRPO adulterated with canola oil; (**B3**) score plot of PLS regression of HRPO adulterated with almond oil; (**C1**) plot of predicted versus actual composition of HRPO adulterated with vegetable oil; (**C2**) plot of predicted versus actual composition of HRPO adulterated with canola oil; (**C3**) plot of predicted versus actual composition of HRPO adulterated with almond oil.

The result of the PLS regression models developed for adulterated HRPOs using VO, CO, and AO adulterants using a full cross validation is shown in Figure 3. In Figure 3, plots A1, A2, and A3 illustrate the regression coefficients as a function of wavenumber for the PLS regression models constructed for adulterated HRPO with VO, CO, and AO adulterant, respectively. The contribution of the magnitude of the coefficients according to wavenumber varies widely. Some wavenumbers contributed positively to the PLS regression, while other wavenumbers contributed negatively to the PLS regression model. The *score plot* of PLS1 versus PLS2 is shown in Figure 3B. The number of the adulterated HRPO samples (*n* = 25 in this study) used for the training set and calibration samples is small in comparison with the FTIR spectral data points (>3600). In theory (*n* − 1), PLSs can be used for data analysis, therefore 24-PLSs can be used in this study. However, the first two PLSs accounted for 100% of the variability in the FTIR spectral data (*x*-variable) and 97% of the percent composition of adulterated HRPO samples. Thus, 2-PLS components are appropriate to represent the data, thereby significantly reducing the data dimensionality. Interestingly, the *score plots* of PLS showed the grouping of the adulterated HRPO samples into two notable and different categories. The samples containing higher percent compositions of VO in the adulterated HRPOs were conspicuously grouped on the right hand side corner (first and second quadrants) of the *score plot*. In contrast, the samples containing higher percent compositions of HRPO in the adulterated samples were grouped on the left hand side (third and fourth quadrants) of the *score plot*. Figure 3(C1) shows the plot of the actual versus the percent compositions of adulterated HRPO with VO determined by the PLS regression. Obviously, the predicted percentage compositions of adulterated HRPO samples favorably compared with the actual percentage composition of adulterated HRPO of the training set and calibration samples. The outcomes of the PLS regression including the *score plot* of the adulterated HRPO with CO and AO adulterants showed similar pattern recognition data. 

A summary of the developed PLS regression models figures-of-merit including the square correlation coefficients (*R*^2^), limits-of-detection (*LOD*), and limits-of-quantification (*LOQ*), are shown in Table 1. The figures-of-merit of the PLS regressions were incredible, with remarkable linearity (*R*^2^ = 0.994191 or better). The *LOD* and *LOQ* values were calculated as 3 *s/m* and 10 *s/m*, respectively, where *s* is the standard deviation of the FTIR intensity of the blanks and *m* is the slope of the PLS regression calibration curve. The *LOD* ranged between 0.02% *wt/wt* for HRPO adulterated with CO and 0.27 % *wt/wt* for HRPO adulterated with VO, demonstrate the capability of the developed PLS regressions for detection of adulterated HRPO at low levels of adulteration.

### 3.4. Determination of Percentage Compositions of Adulterated HRPO Samples

**Figure 4:**
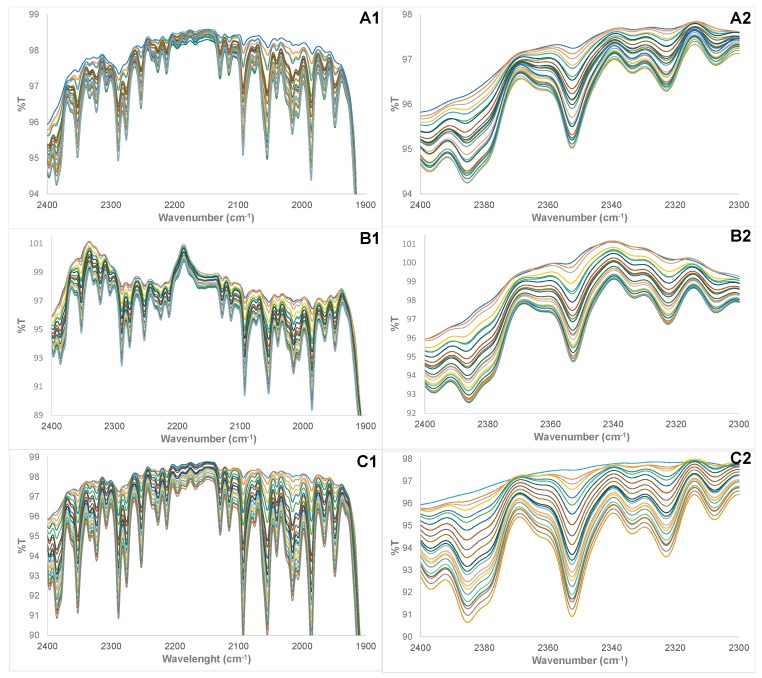
Cross section of FTIR spectra of validation samples of the following: (**A1**,**A2**) HRPO adulterated with vegetable oil, (**B1**,**B2**) HRPO adulterated with canola oil, (**C1**,**C2**) HRPO adulterated with almond oil.

The validation studies were conducted to assess the performance and predictive ability of the PLS regression models for the determination of the percent composition of adulterated HRPO samples. Twenty (22) validation samples each were used for HRPOs that were adulterated with VO and CO. However, 21 validation samples were used for HRPOs adulterated with AO. The FTIR spectra of the adulterated HRPO validation samples using VO, CO, and AO adulterants are shown in Figure 4. It must be highlighted that while the range of the percent compositions of adulterated HRPO in the training set and validation samples are the same, the compositions of adulterated HRPO of the training set and validation samples are totally autonomous. The summary of the results of the validation study conducted for adulterated HRPO showing the actual and the determined compositions of adulterated HRPOs using VO, CO, and AO adulterants are shown in Table 2, Table 3 and Table 4, respectively. The obtained low percent relative error (%RE) of the determined compositions of adulterated HRPOs obviously demonstrates the accuracy of the protocol. The predictive ability of the PLS regression model was further assessed by root-mean-square-relative-percent-errors (*RMS%RE*) for the determination of percent compositions of adulterated HRPOs. The PLS regression models determined percent compositions of adulterated HRPO with VO, CO, and AO with low *RMS%RE* of determination of 2.77%, 5.51%, and 8.32%, respectively, with an overall average *RMS%RE* of 5.53%. 

Although AO is relatively more expensive than HRPO, our study has demonstrated that the purity, authenticity, and percent compositions of adulterated HRPOs can be accurately determined regardless of the edible oil used as adulterant. It must be highlighted that our protocol was not only capable of determining the percentage composition of HRPO in adulterated HRPOs with AO with good accuracy, but it was also capable of determination of the compositions of AO in the adulterated HRPOs with an *RMS%RE* of 4.86% (Table 4). This capability is commendable and appealing, demonstrating the extensive applicability of the protocol for purity analysis of a wide range of edible oils of high dietary and market values.

In order to assess the robustness and reliability of the developed PLS regressions for the determination of percent compositions of future samples of adulterated HRPOs, a set of newly prepared adulterated HRPO samples was prepared over a period of two months. The FTIR spectra of the samples were collected and the originally developed PLS regressions were used to predict the percent compositions of adulterated HRPOs. Interestingly, the developed PLS regression models continued to predict the compositions of newly prepared adulterated HRPOs over a period of two months with incredible accuracy without the need for re-calibration, indicating the robustness of the protocol for purity analysis of adulterated HRPOs.

The result of the study is adoptable and can possibly be used by municipal health departments and local enforcement agencies for rapid, in situ, and field screening of a suspected adulterated HRPO. For instance, the FTIR spectra of pure and adulterated HRPOs can be collected and stored in the database. Hand-held IR spectrometers can be used in situ on the field to rapidly obtain an IR spectrum of a suspected adulterated HRPO. The FTIR spectrum profile of a suspected adulterated HRPO can then be compared with the FTIR spectrum of the adulterated HRPO in the database for similarities or differences. The obtained FTIR spectrum of the adulterated HRPO can be subjected to PLS regression on a laptop computer in the field and optimized. The location of the suspected adulterated HRPOs on the PLS regression *score plot* can be further used for rapid pattern recognition. The developed PLS regression can subsequently be used for purity analysis of the suspected adulterated HRPO samples on the field.

## 4. Conclusions

The result of the combined use of Fourier transform infrared spectroscopy and multivariate partial-least-square (PLS) regression models for rapid purity analysis of highly refined peanut oils (HRPO) that were adulterated with either vegetable oil (VO), canola oil (CO), or almond oil (AO) for food quality assurance purposes is reported. The figures-of-merit of the PLS regression models were incredible with desirable linearity, sensitivity, and robustness. The results of the score plots of the PLS regressions illustrate pattern recognition of the adulterated HRPO samples. The PLS regression models determined compositions of adulterated HRPO with excellent accuracy and low-detection-limits, allowing detection of adulterated HRPO in small quantities. Most importantly, the developed PLS regression models continued to predict the compositions of newly prepared adulterated HRPOs over a period of two months with incredible accuracy without the need for re-calibration, indicating the robustness of the protocol for purity analysis of adulterated HRPOs. The low-cost, non-destructive property; the small sample requirement, high accuracy, and sensitivity; and the simplicity of the protocol make it appealing for quick, in situ, and field screening of suspected adulterated oils by municipalities, health departments, and local enforcement agencies for quality assurance and safety of consumable products.

## Figures and Tables

**Table 1:** Figures-of-merit of partial least squares (PLS) regression calibration curves.

	Wavenumber (cm^−1^)	Offset	Slope	*R*^2^	*LOD* (%*wt/wt*)	*LOQ* (%*wt/wt*)
HRPO-VO	2235–3300	0.572672	0.988415	0.994191	0.27%	0.90
HRPO-CO	2235–3300	0.075944	0.998477	0.999238	0.02%	0.05
HRPO-AO	400–4000	0.154691	0.996644	0.998321	0.02%	0.07

*R^2^—correlation coefficients; LOD—limits-of-detection; LOQ—limits-of-quantification. HRPO—highly refined (100%) peanut oil; VO—vegetable oil; CO—canola oil; AO—almond oil*.

**Table 2:** Validation conducted for highly refined peanut oil (HRPO) adulterated with vegetable oil (VO).

Sample	% HRPO Predicted	Actual % HRPO	%RE	% VO Predicted	Actual % VO	%RE
V1	90.8	89.1	−1.95	9.2	10.9	15.9
V2	85.6	85.1	−0.58	14.4	14.9	3.30
V3	82.5	82.4	−0.07	17.5	17.6	0.33
V4	77.8	79.1	1.63	22.2	20.9	−6.19
V5	72.0	74.0	2.62	28.0	26.0	−7.45
V6	69.2	69.8	0.88	30.8	30.2	−2.02
V7	63.8	64.4	1.04	36.3	35.6	−1.88
V8	61.1	60.4	−1.12	38.9	39.6	1.72
V9	56.5	57.7	1.98	43.5	42.3	−2.69
V10	56.0	54.4	−2.90	44.0	45.6	3.47
V11	52.2	51.6	−1.06	47.8	48.4	1.13
V12	49.3	48.4	−1.96	50.7	51.6	1.84
V13	46.2	45.1	−2.51	53.8	54.9	2.06
V14	43.0	42.7	−0.87	57.0	57.3	0.65
V15	38.6	39.7	2.53	61.4	60.3	−1.66
V16	37.0	37.8	1.94	63.0	62.2	−1.18
V17	34.5	35.8	3.67	65.5	64.2	−2.04
V18	31.4	32.8	4.29	68.6	67.2	−2.09
V19	27.7	29.7	6.76	72.3	70.3	−2.85
V20	27.2	27.2	−0.23	72.8	72.8	0.09
V21	22.9	23.5	2.51	77.1	76.5	−0.77
V22	19.6	20.8	5.79	80.4	79.2	−1.52
**RMS%RE**			2.77			4.37

RE—relative error.

**Table 3:** Validation conducted for highly refined peanut oil (HRPO) adulterated with canola oil (CO).

Sample	% HPPO Predicted	Actual % HPPO	%RE	% CO Predicted	Actual % CO	%RE
V1	89.8	87.9	−2.07	10.2	12.1	15.1
V2	84.0	84.3	0.37	16.0	15.7	−2.01
V3	80.2	82.7	2.96	19.8	17.3	−14.1
V4	77.9	77.2	−0.91	22.1	22.8	3.08
V5	71.0	74.0	4.03	29.0	26.0	−11.5
V6	68.7	70.8	3.00	31.3	29.2	−7.29
V7	63.7	64.3	1.04	36.3	35.7	−1.88
V8	60.5	61.1	0.88	39.5	38.9	−1.38
V9	55.5	58.0	4.36	44.5	42.0	−6.03
V10	54.6	56.0	2.45	45.4	44.0	−3.12
V11	51.5	51.2	−0.68	48.5	48.8	0.71
V12	49.4	48.4	−2.09	50.6	51.6	1.96
V13	46.2	46.4	0.38	53.8	53.6	−0.33
V14	46.3	43.5	−6.45	53.7	56.5	4.97
V15	40.0	41.0	2.31	60.0	59.0	−1.61
V16	37.7	38.5	1.98	62.3	61.5	−1.24
V17	33.8	36.9	8.47	66.2	63.1	−4.96
V18	32.8	34.4	4.64	67.2	65.6	−2.43
V19	29.6	31.5	5.89	70.4	68.5	−2.70
V20	26.3	28.6	8.07	73.7	71.4	−3.23
V21	26.0	25.0	−3.96	74.0	75.0	1.32
V22	20.8	20.9	0.66	79.2	79.1	−0.17
**RMS%RE**			5.51			5.87

**Table 4:** Validation conducted for highly refined peanut oil (HRPO) adulterated with almond oil (AO).

Sample	% HRPO Predicted	Actual % HRPO	%RE	% AO Predicted	Actual % AO	%RE
V1	89.6	88.3	−1.50	10.4	11.7	11.3
V2	85.7	85.9	0.24	14.3	14.1	−1.44
V3	82.3	83.7	1.66	17.7	16.3	−8.54
V4	77.9	79.8	2.45	22.1	20.2	−9.71
V5	76.0	76.7	0.89	24.0	23.3	−2.92
V6	70.3	71.4	1.51	29.7	28.6	−3.76
V7	62.1	64.9	4.25	37.9	35.1	−7.85
V8	58.4	58.7	0.43	41.6	41.3	−0.62
V9	51.3	51.8	0.99	48.7	48.2	−1.06
V10	46.3	44.4	−4.17	53.7	55.6	3.33
V11	39.8	42.5	6.47	60.2	57.5	−4.79
V12	37.3	36.9	−0.97	62.7	63.1	0.57
V13	34.1	33.0	−3.56	65.9	67.0	1.75
V14	28.1	29.4	4.22	71.9	70.6	−1.75
V15	28.1	23.2	−20.77	71.9	76.8	6.29
V16	23.5	20.9	−12.34	76.5	79.1	3.27
V17	21.2	17.7	−19.46	78.8	82.3	4.19
V18	16.8	16.3	−3.13	83.2	83.7	0.61
V19	14.2	14.7	3.42	85.8	85.3	−0.59
V20	11.5	12.3	6.51	88.5	87.7	−0.91
V21	8.3	10.0	17.42	91.7	90.0	−1.95
**RMS%RE**			8.32			4.86
